# Physician Agreement and Confidence in Rural Alaska Medevac Decisions: A Cross-Sectional Vignette Study

**DOI:** 10.64898/2026.09.21.26363599

**Published:** 2026-09-23

**Authors:** Chelsea Williams, Stephanie Weter, Jeremy Wood, Henry Saul, Brian Rice

## Abstract

**Purpose:**

Rural Alaska relies on air ambulances (“medevacs”) to connect remote villages off the road system to higher-level care. Yet medevac decision-making is complicated by limited guidelines, high costs, and delayed transport. We aimed to quantify variability in physician medevac decisions and identify contributing factors.

**Methods:**

We conducted a cross-sectional survey of physicians (n=20) within the Alaska Tribal Health System using 20 clinical vignettes representing varying levels of transport complexity. Participants selected transport modality (medevac, commercial, or remain in village) and rated confidence. Interrater agreement was assessed using Gwet’s AC1. A generalized linear mixed model evaluated predictors of medevac use.

**Findings:**

Agreement was “almost perfect” for clearly critical cases (“always medevac,” AC1=0.97) but declined substantially for intermediate scenarios (“possible medevac” AC1=0.39; “never medevac” AC1=0.26; “special considerations” AC1=0.12). Confidence strongly correlated with agreement (r=0.83, p<0.001), forming distinct clusters of high-, medium-, and low-certainty decisions. Vignette type was the dominant driver of medevac use (OR 455.5 for “always medevac”), but decisions did not change with experience (OR=0.99, 0.94-1.04). Between-physician variability persisted after adjustment (ICC 3.8%; MOR 1.67), consistent with differences in risk tolerance.

**Conclusions:**

Medevac decision-making is highly consistent in certain clinical cases but interrater agreement is limited for intermediate cases. Without a shared gold standard of “correct” medevac decisions, future clinical decision support cannot rely on automated medevac decision making. Efforts to improve care should focus on deconstructing decisions, enhancing outcome feedback, and developing targeted decision support rather than prescriptive decision models.

## Full Text Availability

The license terms selected by the author(s) for this preprint version do not permit archiving in PMC. The full text is available from the preprint server.

